# Hepatocellular Carcinoma Targeted Thermosensitive Cu^2+^ Liposomes Based on the Cuproptosis Mechanism

**DOI:** 10.1002/mco2.71068

**Published:** 2026-10-13

**Authors:** Hao Yang, Hui Hou, Hong Li, Xianglong Wang, Wei Zhang, Mengsi Yin, Fuyu Zhang, Faxin Zhang, Mingyuan Li, Xiangcheng Wang, Xianggao Zhu

**Affiliations:** ^1^ Department of Radiation Oncology Peking University Cancer Hospital (Inner Mongolia Campus) & Affiliated Cancer Hospital of Inner Mongolia Medical University Inner Mongolia Autonomous Region Hohhot China; ^2^ Department of Pediatric Maternal and Child Health Care Hospital of the Inner Mongolia Autonomous Region Inner Mongolia Autonomous Region Hohhot China; ^3^ Department of Pediatric Hematology and Oncology Inner Mongolia Autonomous Region People's Hospital Inner Mongolia Autonomous Region Hohhot China; ^4^ College of Biotechnology Tianjin University of Science & Technology Tianjin China; ^5^ Department of Nuclear Medicine Shenzhen People's Hospital Shenzhen China

**Keywords:** cuproptosis, galactose modification, hepatocellular carcinoma, targeted therapy, thermosensitive liposome

## Abstract

Cuproptosis is characterized by elevated intracellular copper concentrations that disrupt copper homeostasis and impair the tricarboxylic acid cycle, ultimately causing cell death. However, the lack of targeted intervention for intracellular copper may induce systemic side effects, hindering the application of cuproptosis in tumor therapy. Herein, we constructed a targeted copper ion delivery system (Cu‐Lip‐Gal(H)) based on galactose‐modified thermosensitive liposomes, combined with local tumor hyperthermia targeted copper delivery into hepatocellular carcinoma (HCC) cells. Cu‐Lip‐Gal showed excellent physicochemical properties and 42°C thermosensitive release, with 64.67% ± 2.52% cumulative release within 0.5 h. The Cu‐Lip‐Gal(H) group reduced HepG2 and Huh7 cell viability to 24.48% ± 3.31% and 18.00% ± 3.18%, respectively; it exhibited 5.8‐fold higher intracellular copper concentration in HepG2 cells and 5.2‐fold higher in Huh7 cells. In nude mouse xenografts, Cu‐Lip‐Gal(H) achieved a tumor inhibition rate of 86.7% ± 1.9% without obvious histopathological changes in major organs but significantly increased necrotic cells in tumors along with the highest FDX1 expression levels observed in this study. These findings provide new insights into cuproptosis‐based copper ion delivery systems for HCC treatment.

## Graphical abstract

This study develops galactose‐modified thermosensitive liposomes loaded with copper ions for the treatment of hepatocellular carcinoma. Combined with local tumor hyperthermia, this system delivers copper ions into tumor cells, induces cuproptosis by regulating FDX1 and DLAT expression and disrupting ROS/NADPH homeostasis, and suppresses tumor progression in cellular and animal models.

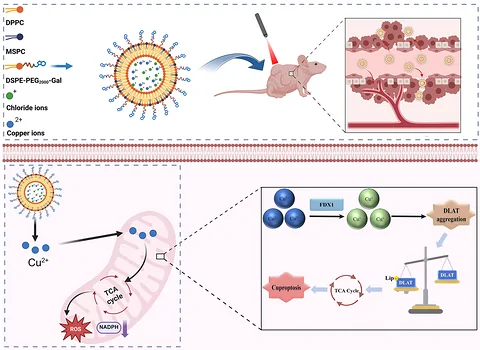

## 1 Introduction

Liver cancer is the sixth most common cancer worldwide and the fourth leading cause of cancer‐related death, with a 5‐year survival of 18% [1, 2]. Hepatocellular carcinoma (HCC) accounts for 90% of the cases [3]. Surgery remains the primary treatment for HCC, but the resection is indicated in only 5%–15% of cases and is only suitable for early‐stage disease [4, 5, 6, 7]. Conventional chemotherapy lacks targeting specificity and has strong systemic toxicity generally [8, 9, 10].

As an essential micronutrient, copper is required for a wide range of physiological processes in virtually all cell types. While copper ions act as indispensable cofactors for numerous enzymes and are pivotal in various metabolic processes, disturbances in copper ion homeostasis can have detrimental effects on biological functions [11, 12]. The intracellular Cu concentration is maintained within a relatively low range because the uptake, distribution, and elimination of Cu are tightly regulated to maintain copper homeostasis [13]. Notably, the inability to maintain Cu balance has been associated with a wide range of pathological conditions, including Menkes disease, Wilson disease, neurodegenerative diseases, and cardiovascular disease [14]. Thus, systemic Cu levels must be maintained within a narrow range to ensure normal biochemical processes.

Cuproptosis is a recently discovered form of regulated cell death triggered by excess Cu^2+^. This Cu‐induced programmed cell death is mechanistically distinct from other well‐characterized cell death pathways, such as apoptosis or ferroptosis [15]. Apoptosis is a caspase‐dependent pathway driven by the activation of death receptors or mitochondrial outer membrane permeabilization, characterized by chromatin condensation, nuclear fragmentation, and the formation of apoptotic bodies without disrupting metabolic homeostasis [16, 17]. Ferroptosis is an iron‐dependent form of cell death mediated by the accumulation of lipid peroxides, which is tightly linked to glutathione (GSH) depletion and inactivation of glutathione peroxidase 4 (GPX4), with no direct involvement in mitochondrial metabolic enzymes [18, 19].

In contrast, cuproptosis is initiated by the direct binding of intracellular Cu^2+^ to lipoylated components of the tricarboxylic acid (TCA) cycle (DLAT), leading to the aggregation of these Cu‐bound lipoylated mitochondrial proteins. This aggregation further impairs the function of iron–sulfur (Fe‐S) clusters, induces proteotoxic stress, and ultimately disrupts mitochondrial metabolism to drive cell death [14]. Given its roles in cancer proliferation, angiogenesis, and metastasis, copper ion‐induced cuproptosis has been considered to hold great potential for cancer treatment. Copper ionophores are copper‐binding small molecules that shuttle copper into cells and are thereby useful tools for inducing cuproptosis [20, 21]. However, when exploiting the cuproptosis mechanism for tumor treatment, targeted delivery of Cu^2+^ to tumor cells without disturbing copper balance in normal cells has become a bottleneck issue [19, 22].

Nanomedicine may offer an effective targeted therapeutic approach to prolong drug circulation, modulate drug release, improve in vivo efficacy, and reduce off‐target effects [23, 24, 25]. Thermosensitive liposomes (TSLs) were developed and pursued in combination with localized hyperthermia (HT) at the tumor site [26, 27]. TSLs afford rapid drug release once heated to mild HT temperatures (39°C–43°C) in the tumor vasculature, which is achievable by using an external microwave or high‐intensity focused ultrasound [28, 29, 30, 31, 32]. Owing to the enhanced permeability retention (EPR) effect, TSLs (about 80–150 nm) first accumulate in solid tumors; mild HT then triggers drug release, creating a local concentration gradient that drives drug penetration into surrounding tumor tissue, including the tumor boundary [33, 34, 35, 36, 37].

Additionally, tumor cell sensitivity increases and DNA repair activity is inhibited during HT [38]. Galactose is the most widely used ligand for liver‐directed drug delivery because its receptor, asialoglycoprotein (ASGPR), is exclusively expressed on the surface of hepatocytes and hepatoma cells with high density [39, 40]. ASGPR can recognize terminal β‐D‐galactose residues with high specificity and efficiency. The decoration of galactose residues on liposomes can enhance drug internalization in hepatocytes via ligand‐receptor‐mediated endocytosis [40, 41, 42, 43].

In this study, we developed galactose‐modified TSLs encapsulating Cu^2+^ (Cu‐Lip‐Gal) in combination with HT for targeted liver cancer therapy by leveraging the cuproptosis mechanism. Specifically, we aimed to capitalize on the 100 nm particle size of liposomes to achieve passive targeting via the EPR effect characteristic of solid tumors. Galactose was employed as a targeting moiety for active targeting, enabling specific binding to liver cancer cells. Additionally, thermosensitive release was integrated with locally controllable HT for physicochemical targeting. Through this targeting approach, Cu^2+^ was precisely delivered into liver cancer cells, thereby inhibiting tumor proliferation via the cuproptosis mechanism. Our precise‐targeting delivery system offers a novel strategy for the application of cuproptosis in tumor treatment, potentially addressing the bottleneck issues associated with its clinical translation.

## 2 Results

### 2.1 Characterization of Cu‐Lip‐Gal

Colloids of 10–100 nm exhibit a Tyndall effect under laser irradiation (Figure 1A), while molecular solutions do not [44, 45]. Transmission electron microscopy (TEM) further confirmed that both formulations were monodisperse, smooth‐surfaced spheres (Figure 1B), with DLS showing hydrodynamic diameters of 113.8 ± 2.4 nm (Cu‐Lip, polydispersity index [PDI] 0.22 ± 0.02) and 120.5 ± 3.1 nm (Cu‐Lip‐Gal, PDI 0.15 ± 0.02; Figure 1C,D). Zeta potentials were 11.06 ± 1.62 mV (Cu‐Lip) and 8.08 ± 1.31 mV (Cu‐Lip‐Gal), with encapsulation efficiencies of 64.94% ± 2.67% and 70.72% ± 1.36%, respectively (Figure 1E,F). The slightly larger size of Cu‐Lip‐Gal was due to galactose‐PEG2000 surface modification (Figure 1G), consistent with imaging and sizing data. Stability testing showed that over 14 days at 4°C, both formulations exhibited moderate increases in particle size and zeta potential, with stable PDI confirming colloidal integrity (Figure 1H–J). Incubation in 10% FBS for 3 days resulted in only minor changes in particle size, PDI, zeta potential, and encapsulation efficiency (Figure S1), demonstrating favorable physiological stability critical for in vivo circulation and targeted delivery.

**FIGURE 1:**
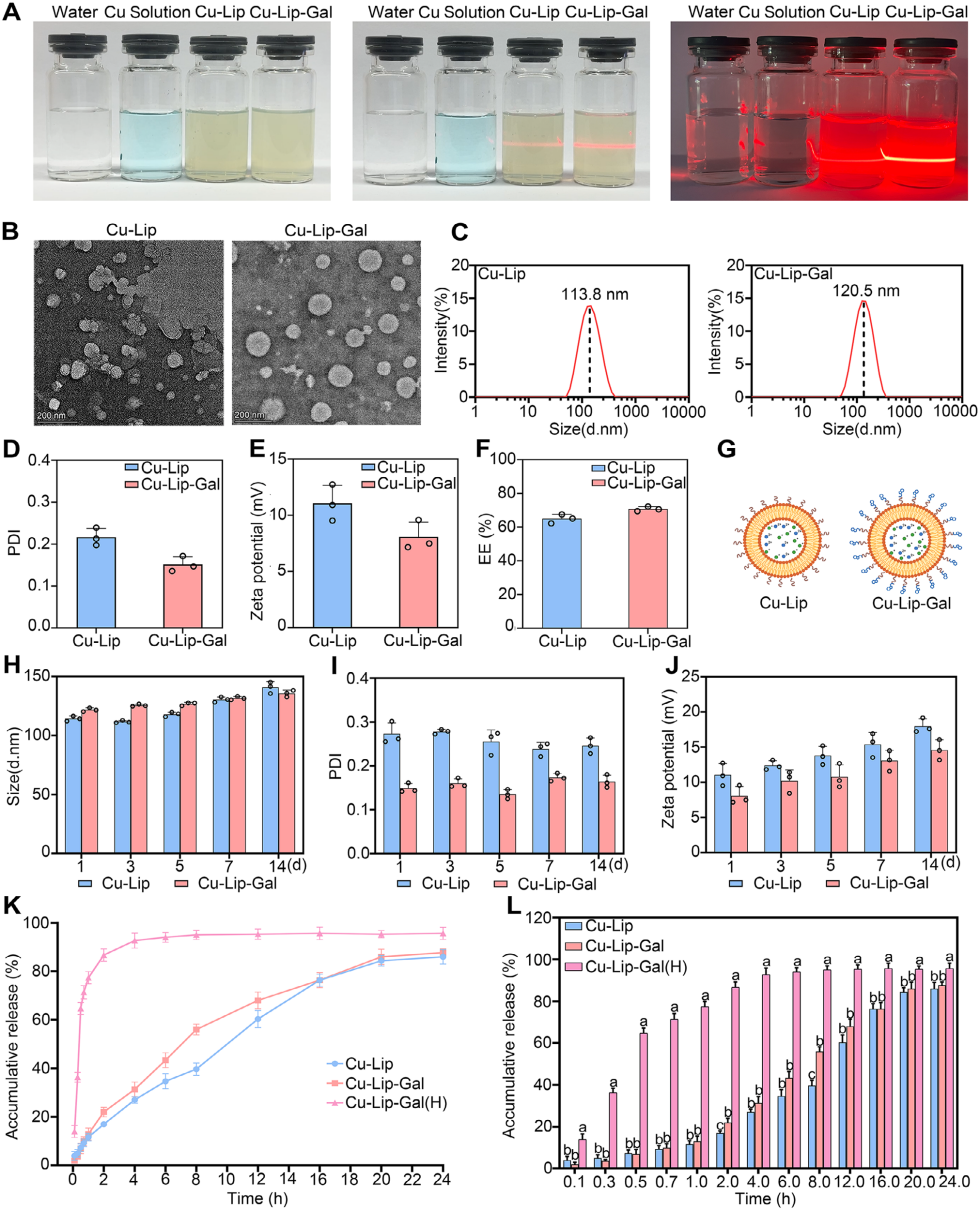
Characterizations of different formulations. (A) Appearance and Tyndall effect. (B) The micromorphology of Cu‐Lip and Cu‐Lip‐Gal observed by TEM. (C) The size distribution. (D) The PDI. (E) The zeta potential. (F) The EE of Cu‐Lip and Cu‐Lip‐Gal. (G) Schematic diagrams of Cu‐Lip and Cu‐Lip‐Gal. (H–J) Time‐dependent changes of size distribution, PDI and zeta potential of Cu‐Lip and Cu‐Lip‐Gal storage 14 days at 4°C. (K) Cumulative release curves of Cu‐Lip, Cu‐Lip‐Gal and Cu‐Lip‐Gal (H) in 24 h. (L) Cumulative release rate of Cu‐Lip, Cu‐Lip‐Gal and Cu‐Lip‐Gal (H) at each time point.

In vitro release assays showed sustained Cu^2^^+^ release at 37°C (86%–88% over 24 h) for both formulations (Figure 1K), while 0.5 h at 42°C triggered rapid burst release from Cu‐Lip‐Gal (64.67% ± 2.52% in 0.5 h), with 95.67% ± 1.53% total release at 24 h. Cu‐Lip‐Gal(H) exhibited significantly higher cumulative release than Cu‐Lip and Cu‐Lip‐Gal at most time points (Figure 1L).

### 2.2 Cell Viability and Cu Content in Cells

The cytotoxicity of Cu^2+^‐loaded liposomes was evaluated by measuring the viability of HepG2 and Huh7 cells using MTT and CCK‐8 assays (Figure 2A–H). Both cell lines were highly sensitive to Cu‐Lip, which encapsulated and delivered Cu^2+^ into cells far more effectively than Cu^2+^ solution. Cu‐Lip‐Gal featured galactose modification on the surface of Cu‐Lip, enabling binding to ASGPR receptors on HepG2 and Huh7 cells and thereby further enhancing delivery efficiency. For the Cu‐Lip‐Gal(H) group, heating at 42°C increased membrane fluidity, thereby enabling maximal intracellular delivery of Cu^2+^. Regarding inhibition of cell proliferation, cuproptosis and HT‐induced impairment of DNA repair exhibited a synergistic effect. Notably, to verify the selective cytotoxicity of our formulations, we performed cytotoxicity assays on normal human hepatocytes (LO2 cells) at the same Cu^2+^ concentration (2.5 µM); the results (Figure S2) showed that LO2 cells maintained high viability after treatment with Cu‐Lip‐Gal(H) (MTT assay: 76.42% ± 2.35%; CCK‐8 assay: 73.65% ± 1.90%), with no significant growth inhibition observed in the 48‐h proliferation assay. This starkly contrasts with the profound reduction in viability of HepG2 cells (MTT assay: 24.48% ± 3.31%; CCK‐8 assay: 25.50% ± 2.52%) and Huh7 cells (MTT assay: 18.00% ± 3.18%; CCK‐8 assay: 15.96% ± 2.68%) under identical conditions, confirming the biological safety of Cu‐Lip‐Gal(H) toward normal hepatocytes.

**FIGURE 2:**
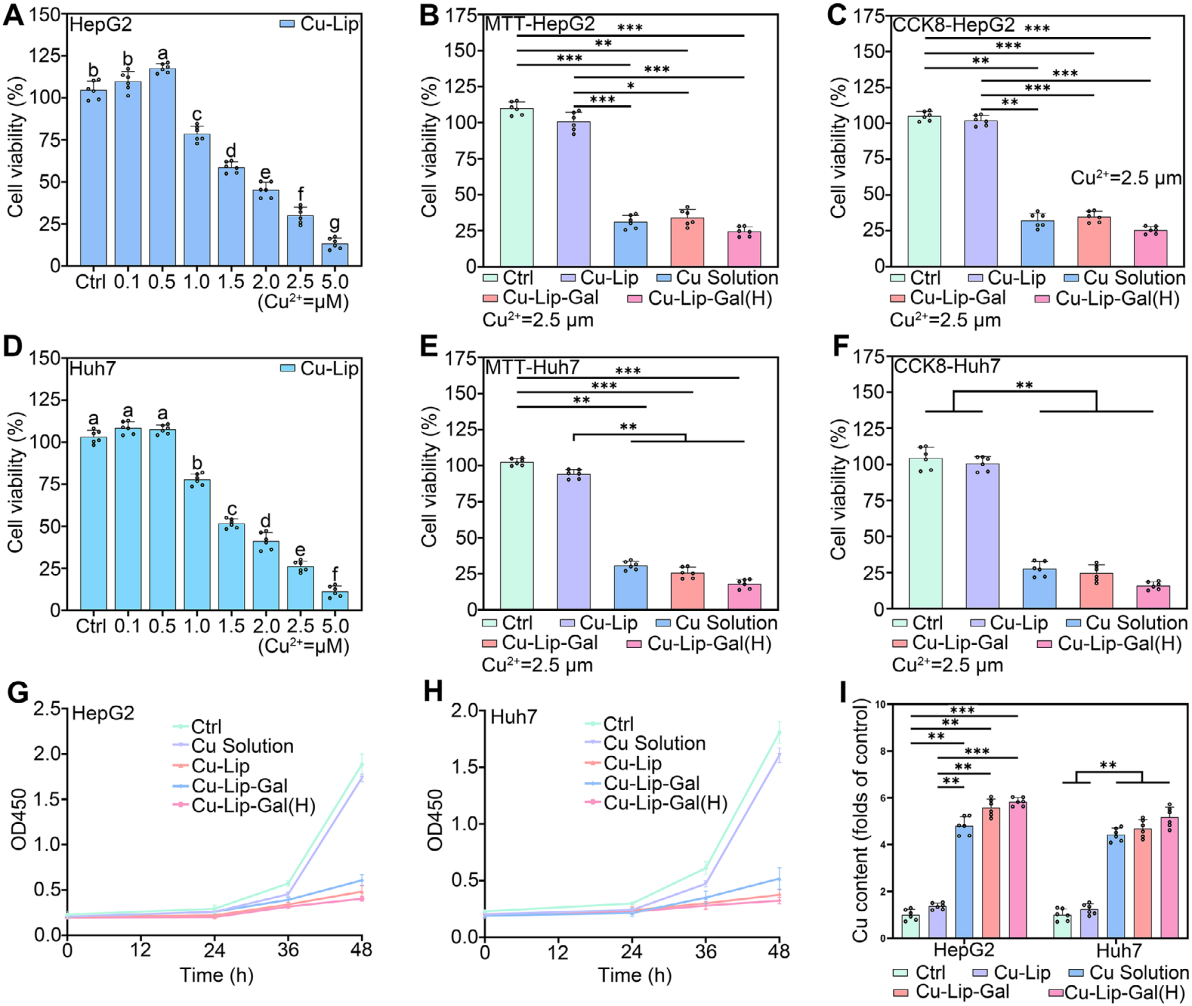
Cell viability and Cu content in HepG2 and Huh7 cells. (A) MTT assay for HepG2 cell treated with Cu‐Lip of different concentration. The cell viability of HepG2 cell treated with phosphate‐buffered saline (PBS), Cu solution, Cu‐Lip, Cu‐Lip‐Gal and Cu‐Lip‐Gal (H): (B) MTT assay; (C) CCK8 assay. (D) MTT assay for Huh7 cell treated with Cu‐Lip of different concentration. The cell viability of Huh7 cell treated with PBS, Cu solution, Cu‐Lip, Cu‐Lip‐Gal and Cu‐Lip‐Gal (H): (E) MTT assay; (F) CCK8 assay. (G) The HepG2 cell growth curves after treatments in 48 h. (H) The Huh7 cell growth curves after treatments in 48 h. (I) The Cu content in HepG2 and Huh7 cells after treatments (calculated as a multiple of the control group).

Through the determination of intracellular copper content (Figure 2I), the Cu content in the Cu solution group was only 1.4‐fold that of the control group in HepG2 cells (1.2‐fold in Huh7 cells). This indicated that, under the protection of the cell membrane, intracellular copper homeostasis was not readily perturbed and that Cu^2^^+^ did not readily enter cells via simple diffusion. In contrast, the liposome groups significantly enhanced copper uptake: intracellular copper content reached 4.8‐fold (HepG2) and 4.4‐fold (Huh7) that of the control in the Cu‐Lip group. The Cu‐Lip‐Gal(H) group showed the highest accumulation, with 5.8‐fold in HepG2 and 5.2‐fold in Huh7 relative to the control, consistent with its superior cytotoxicity profile. These results suggested that the decrease in cell viability was closely related to the increase in intracellular Cu content, indicating that cuproptosis was triggered to varying degrees.

### 2.3 The Cell Uptake Pathways of Cu‐Lip‐Gal

To characterize the cell uptake pathways of Cu‐Lip‐Gal, its uptake behavior in HepG2 cells was observed by confocal laser scanning microscopy (CLSM). As shown in Figure 3A, Cu‐Lip‐Gal/Cou‐6 exhibited green, while the cell nucleus showed blue. The cellular uptake mechanism was elucidated by comparing green fluorescence intensity under different inhibitory conditions targeting specific pathways: chlorpromazine (clathrin‐mediated endocytosis inhibitor), colchicine (macropinocytosis inhibitor), and incubation at 4°C (ATP‐dependent process inhibitor). The control group demonstrated efficient uptake of Cu‐Lip‐Gal/Cou‐6 by HepG2 cells, and endocytosis inhibitor groups exhibited varying degrees of inhibitory effects. Figure 3B revealed that the 4°C group exhibited the lowest fluorescence intensity, indicating that active ATP‐dependent mechanisms played a central role in Cu‐Lip‐Gal cellular uptake. Both clathrin‐mediated endocytosis and macropinocytosis were cellular uptake pathways, among which clathrin‐mediated endocytosis was more predominant.

**FIGURE 3:**
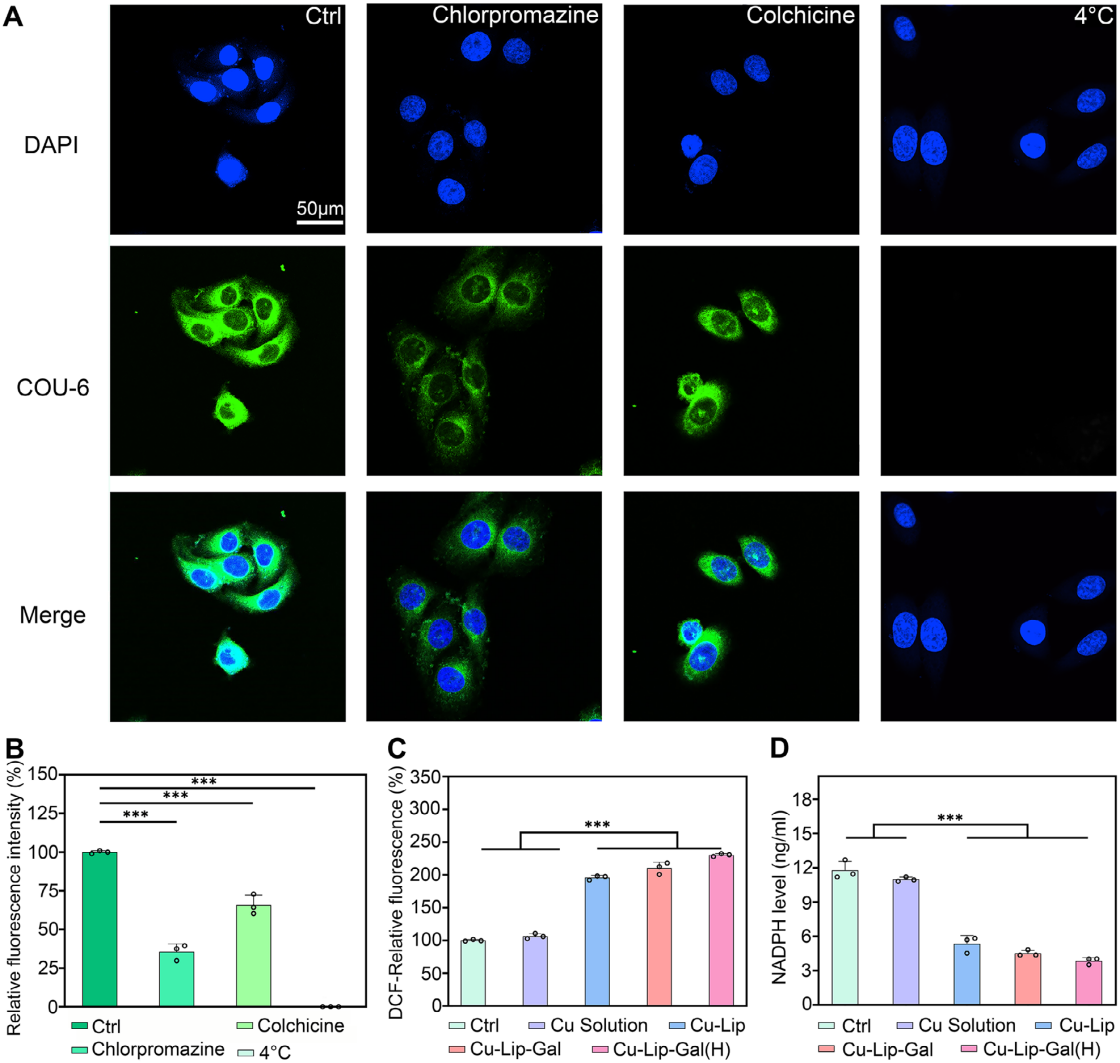
Cellular uptake pathways of Cu‐Lip‐Gal and its effects on intracellular redox homeostasis in HepG2 cells. (A) Cell uptake for Cu‐Lip‐Gal under different inhibition conditions captured by confocal laser scanning microscopy (CLSM). (B) Relatively fluorescence intensity of each inhibition conditions. (C) ROS levels in HepG2 cells after different treatments. (D) NADPH level in HepG2 cells after different treatments.

### 2.4 The Intracellular NADPH/ROS Level and Oxidative Stress Intervention

To elucidate the cuproptosis‐inducing mechanism of Cu‐loaded liposomes, we assessed intracellular NADPH and ROS levels in HepG2 cells. Quantitative analysis revealed that treatment with Cu‐Lip, Cu‐Lip‐Gal, and Cu‐Lip‐Gal(H) significantly elevated intracellular ROS levels to approximately 2.0‐, 2.1‐, and 2.3‐fold of the control, respectively (Figure 3C). Concurrently, NADPH levels were markedly depleted, showing reductions of approximately 51.1% ± 3.7%, 61.5% ± 4.4%, and 67.1% ± 4.5%, compared to the control group (Figure 3D). While cuproptosis primarily involves copper‐dependent aggregation of lipoylated TCA cycle proteins and proteotoxic stress, our findings reveal that this process was accompanied by profound metabolic perturbations, including ROS accumulation and NADPH depletion. The Cu loaded liposomes (Cu‐Lip, Cu‐Lip‐Gal and Cu‐Lip‐Gal(H))—but not free Cu^2+^—triggered a near doubling of ROS levels, consistent with copper‐induced mitochondrial dysfunction, impaired TCA cycle activity, and electron leakage leading to superoxide production. To further verify the causal role of oxidative stress in Cu‐Lip‐Gal(H)‐induced cuproptosis, an N‐acetylcysteine (NAC)‐based intervention experiment was performed (Figure S3). The analysis revealed that Cu‐Lip‐Gal(H) treatment markedly disrupted the intracellular redox balance in HepG2 and Huh7 cells. Compared with the control group, intracellular ROS levels increased to 2.21 ± 0.10‐fold and 2.05 ± 0.11‐fold, respectively, while relative NADPH levels decreased significantly by 62.6% ± 3.1% and 59.0% ± 3.4%. In sharp contrast, pre‐incubation with NAC effectively abolished these redox imbalances. In NAC + Cu‐Lip‐Gal(H)‐treated HepG2 and Huh7 cells, ROS levels were only 1.24 ± 0.09‐fold and 1.13 ± 0.11‐fold of the control, respectively, and the depleted NADPH levels were substantially restored to 81.4% ± 2.4% and 88.5% ± 5.5% of the control. Concurrently, NAC pretreatment significantly reversed the cytotoxicity of Cu‐Lip‐Gal(H) (Figure S3). These results collectively confirm that oxidative stress acts as a key mediator in Cu‐Lip‐Gal(H)‐triggered cuproptosis in HCC cells.

### 2.5 HepG2 Cell Growth Can Be Suppressed by Cu‐Lip‐Gal Together With Mild HT

Figure 4A illustrates the inhibitory effect of different treatments on the colony‐forming capacity of HepG2 cells. The cell scratch assay was performed to evaluate the inhibitory effects of various treatment groups on the migratory ability of HepG2 cells. As shown in Figure 4B, the degree of scratch wound healing in the Cu‐Lip‐Gal(H) group was significantly lower than that in the other groups at 48 h. Quantitative analysis revealed that the colony formation rate of the Cu‐Lip‐Gal(H) group was reduced by 62.3% ± 4.1%, compared with the Cu‐Lip group (Figure 4C), and the cell migration distance in this group was also significantly shorter than that in the other groups (Figure 4D). Analysis of the relative migration rates (Figure 4E) revealed a similar trend: The Cu‐Lip‐Gal(H) group consistently exhibited the lowest migration rate and the smallest relative increase, indicating its sustained and stable inhibitory efficacy. Collectively, these results indicate a progressive enhancement in the inhibition of tumor cell migration across the Cu‐Lip, Cu‐Lip‐Gal, and Cu‐Lip‐Gal(H) groups. This supports a potential therapeutic strategy combining Cu‐Lip‐Gal with HT to enhance migration inhibition and reduce metastasis risk by triggering cuproptosis.

**FIGURE 4:**
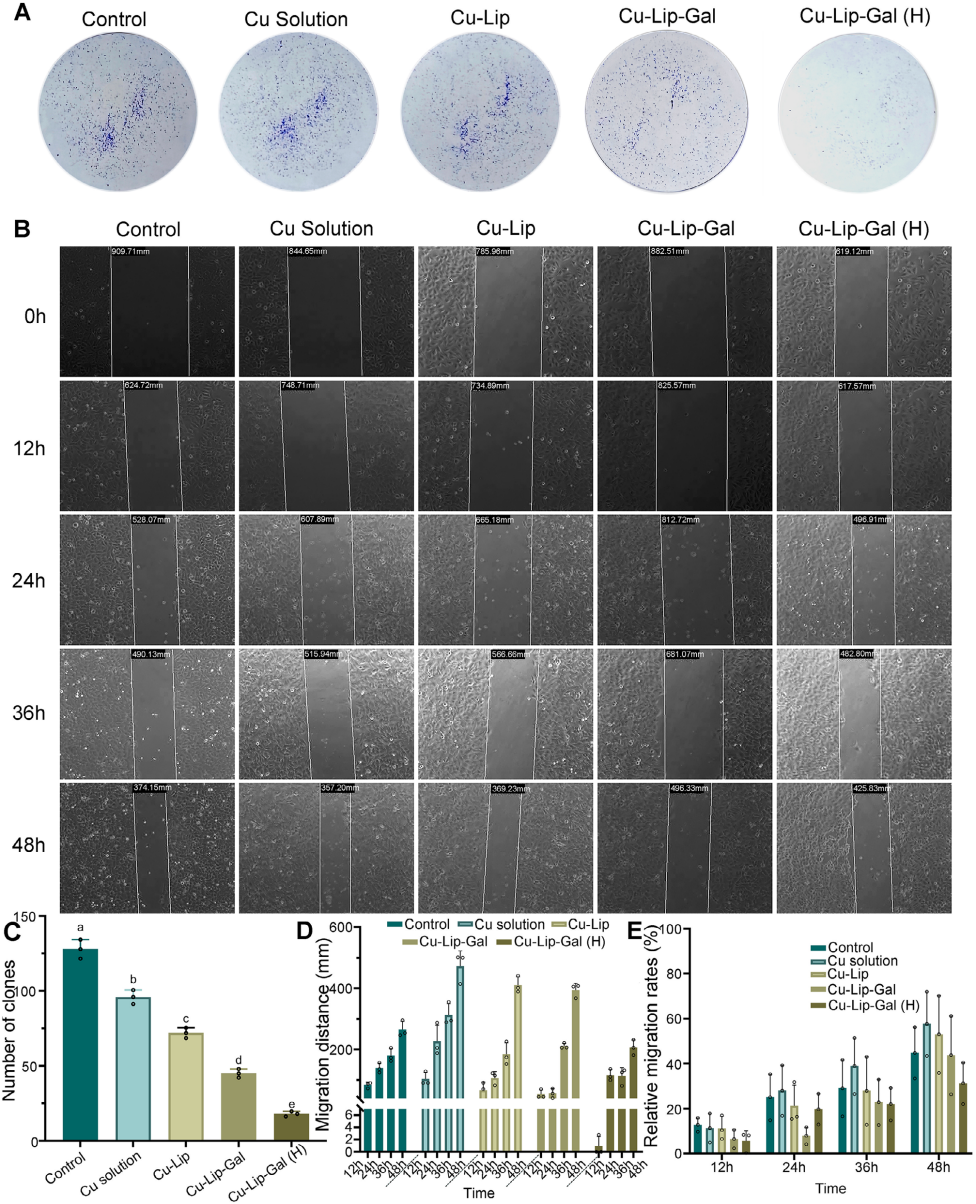
Cu‐Lip‐Gal (H) inhibits the cell growth, proliferation, and migration of HepG2 cells. (A) Colony formation. (B) Scratch assay in 48 h. (C) Number of clones. (D) Cell migration distance. (E) Relative migration rate. (*n* = 3.)

### 2.6 The Synergistic Mechanism of Cu‐Lip‐Gal and HT Enhances Cuproptosis

The cuproptosis mechanism is depicted in Figure 5A. FDX1 and DLAT are key cuproptosis regulators in HepG2 cells under different treatments. DLAT, a pivotal TCA cycle enzyme, undergoes oligomerization during cuproptosis [14]. FDX1 is a core cuproptosis regulator that mediates copper sensing and death signaling [46]. Western blot analysis demonstrated that DLAT was predominantly detected as a monomeric band in cells treated with phosphate‐buffered saline (PBS) or Cu solution (Figure 5B). Quantitative densitometric analysis of band intensities—normalized to tubulin and calibrated against the PBS group (set to 1.00)—revealed that the relative expression of monomeric DLAT in the Cu solution group remained relatively unchanged at 0.92‐fold, compared with the PBS group. By contrast, monomeric DLAT levels exhibited a gradual upward trend in the copper‐loaded liposome groups, reaching 1.46‐fold, 1.76‐fold, and 2.48‐fold of the PBS group in the Cu‐Lip, Cu‐Lip‐Gal, and Cu‐Lip‐Gal(H) groups, respectively (Figure 5C). Consistently, quantitative analysis of FDX1 expression revealed a distinct translational gradient: Its levels increased to 1.58‐fold relative to the PBS group in the Cu solution group and further rose to 2.58‐fold, 2.88‐fold, and 3.17‐fold in the Cu‐Lip, Cu‐Lip‐Gal, and Cu‐Lip‐Gal(H) groups, respectively (Figure 5C). Collectively, these findings indicate that the extent of copper‐triggered cuproptosis in HepG2 cells is positively correlated with the intracellular copper concentration achieved by different delivery systems. This correlation is evidenced by the progressive upregulation of core cuproptosis mediators—including FDX1 and DLAT—and the pronounced induction of DLAT oligomerization in groups with enhanced copper delivery efficiency [47].

**FIGURE 5:**
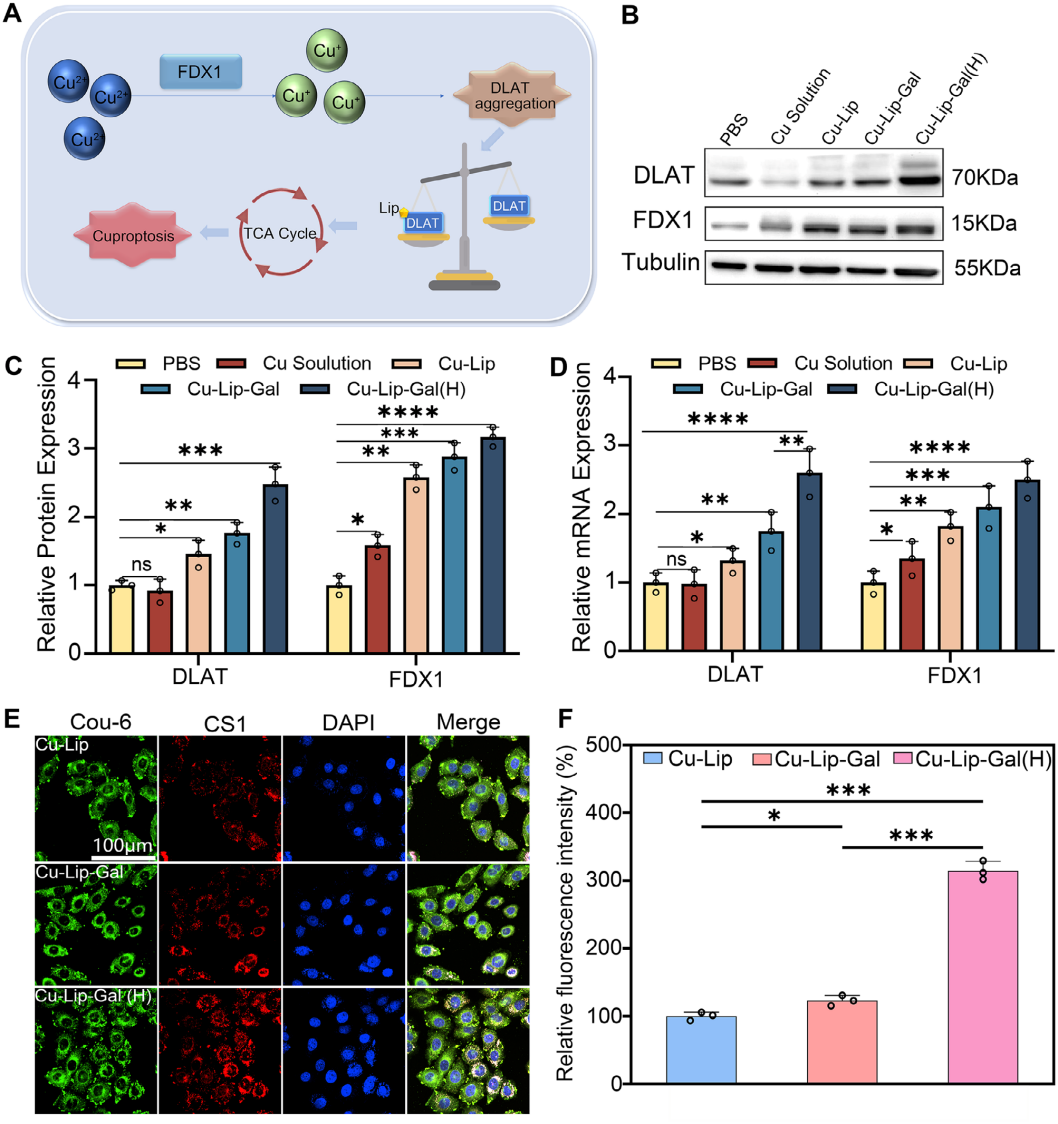
Mechanism of Cu‐Lip‐Gal(H)‐induced cuproptosis in HepG2 cells. (A) The schematic illustration of cuproptosis mechanism. (B) The protein levels of DLAT, FDX1, and Tubulin were detected by western blotting. (C) Relative protein expression levels of DLAT and FDX1 by western blotting. (Monomeric DLAT levels were 0.92‐, 1.46‐, 1.76‐, and 2.48‐fold, while FDX1 levels were 1.58‐, 2.58‐, 2.88‐, and 3.17‐fold in Cu Solution, Cu‐Lip, Cu‐Lip‐Gal, and Cu‐Lip‐Gal(H) groups, respectively. Data are presented as mean ± SD.) (D) DLAT and FDX1 mRNA expression in HepG2 cells was determined by qPCR. (*n* = 3.) (E) Monitoring Cu^+^ levels by CLSM. (F) Relative fluorescence intensity of CS1 in HepG2 cells.

As shown in Figure 5D, qPCR analysis of DLAT and FDX1 mRNA was consistent with the western blotting results. For DLAT mRNA, expression was 0.98‐fold of the PBS level in the Cu solution group, 1.32‐fold in the Cu‐Lip group, and 1.75‐fold in the Cu‐Lip‐Gal group; notably, the Cu‐Lip‐Gal(H) group showed a further significant increase to 2.60‐fold, confirming that HT augments DLAT transcriptional activation. Similarly, FDX1 mRNA was only modestly upregulated (1.35‐fold vs. PBS) in the free Cu group but markedly increased in all copper‐loaded liposome groups, reaching 1.82‐, 2.10‐, and 2.50‐fold of the PBS level in the three liposome groups. These results indicate that efficient intracellular copper delivery via liposomal formulations is essential for robust FDX1 transcriptional upregulation—a critical step in cuproptosis initiation. Intracellular Cu^+^ levels in HepG2 cells were monitored using Copper Sensor 1 (CS1). As shown in Figure 5E,F, CLSM revealed a gradual elevation in red fluorescence intensity in the order of Cu‐Lip < Cu‐Lip‐Gal < Cu‐Lip‐Gal(H), consistent with the stepwise enhancement in Cu^2^^+^ delivery efficiency of our targeting strategy. Notably, Cu‐Lip‐Gal(H) enabled heat‐triggered Cu^2^^+^ release to maximize intracellular Cu^2^^+^ accumulation, which was then efficiently reduced to Cu^+^ in an FDX1‐dependent manner, matching the gradual intensification of CS1 fluorescence. These results corroborate the pivotal role of FDX1‐mediated Cu^2^^+^‐to‐Cu^+^ reduction in cuproptosis progression.

### 2.7 Targeted Cu‐Lip‐Gal (H) Enhanced In Vivo Anti‐Tumor Efficacy

To evaluate the in vivo therapeutic efficacy, a xenograft nude mouse model bearing HepG2 cells was employed. The experimental protocol is illustrated in Figure 6A. As shown in the tumor growth curves (Figure 6B,C), both the control group and the copper solution group exhibited rapid and sustained tumor progression. In contrast, all liposomal formulations significantly suppressed tumor growth (Figure 6D–F). The Cu‐Lip group showed moderate inhibition, whereas the Cu‐Lip‐Gal group demonstrated superior efficacy, compared to Cu‐Lip, highlighting the advantage of galactose‐mediated active targeting HCC cells. Notably, the Cu‐Lip‐Gal(H) group displayed the strongest tumor growth inhibition (inhibition rate: 86.7% ± 1.9%). Body weight monitoring served as a preliminary indicator of systemic toxicity (Figure 6G). Mice in the control group showed the most pronounced weight gain, likely attributable to the rapid increase in tumor burden. Mice treated with copper solution initially exhibited weight stagnation followed by a decline, possibly reflecting systemic copper toxicity manifested as reduced activity and loss of appetite. Remarkably, mice treated with Cu‐Lip‐Gal(H) maintained a consistent increase in body weight, second only to the control group, despite having the lowest tumor burden. This indicates that the targeting strategy of the formulation effectively concentrated the therapeutic effect in the tumor region, minimized systemic exposure and associated adverse effects, and thereby preserved the overall health status of the animals.

**FIGURE 6:**
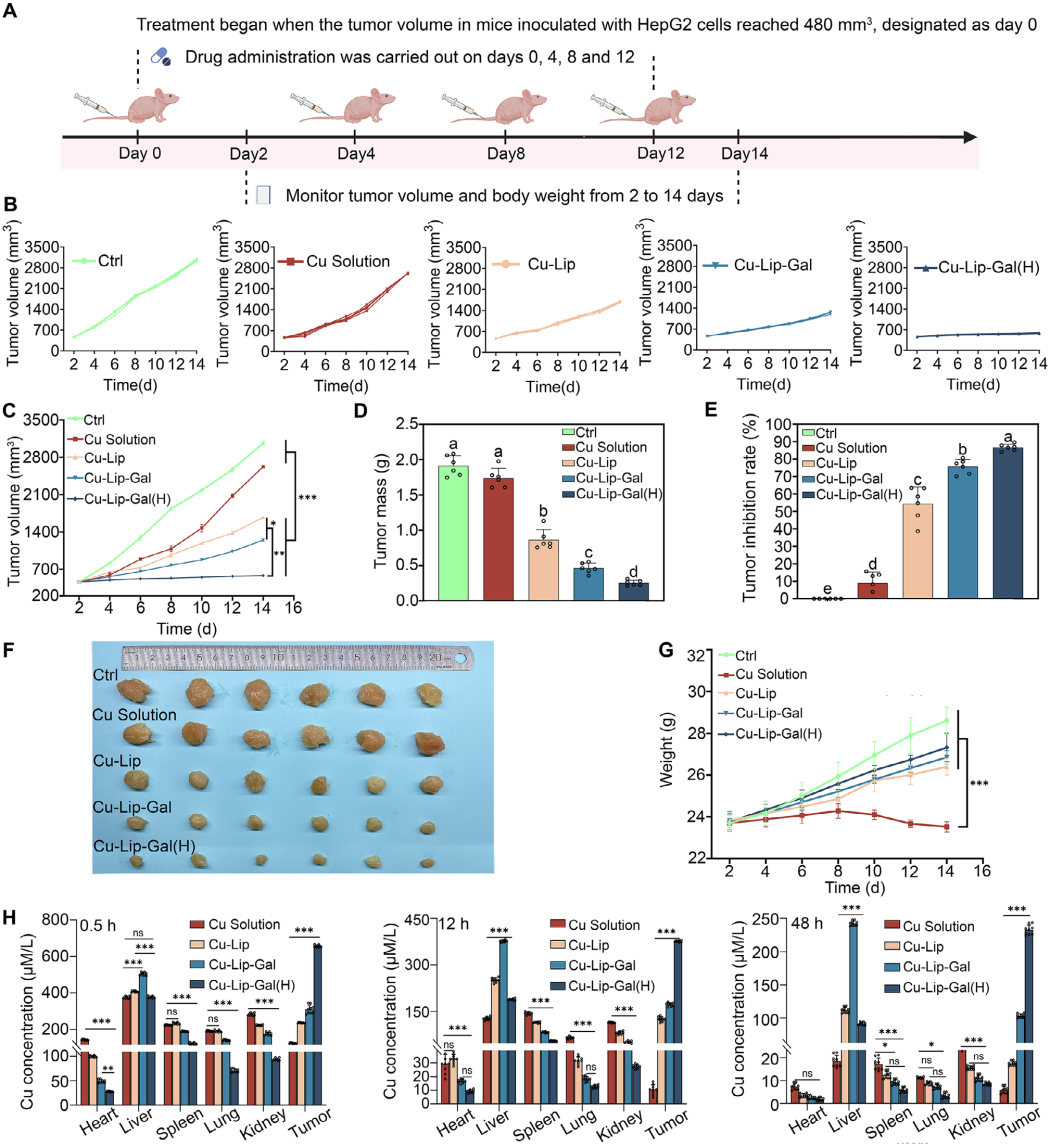
In vivo antitumor efficacy of thermosensitive Cu‐Lip‐Gal(H) (n=6). (A) Schematic illustration of the experimental protocol in HepG2 tumor‐bearing nude mice with different formulations. (B) Tumor volume growth curves after treatment with normal saline (control), Cu Solution, Cu‐Lip, Cu‐Lip‐Gal, and Cu‐Lip‐Gal(H) at a Cu^2+^ dosage of 1.28 mg/kg. (C) Summarized curves of relative tumor volume. (D) Absolute tumor weight. (E) Tumor inhibition rate. (F) Excised tumors from sacrificed mice. (G) Body weight of HepG2 tumor‐bearing nude mice during the treatment period. (H) Copper concentrations in major organs and tumor tissues at 0.5, 12, and 48 h post‐administration.

### 2.8 The Influence of Targeting on the Distribution of Copper In Vivo

As shown in Figure 6H, the Cu distribution across various tissues following administration of different formulations (Cu solution, Cu‐Lip, Cu‐Lip‐Gal, and Cu‐Lip‐Gal(H)) was determined at 0.5, 12, and 48 h. The liver serves as an organ for copper storage [48]. At 0.5 h, the Cu solution group lacked targeting ability, and high levels of copper were detected in various organs, with the highest content in the liver and the lowest in the tumor. All liposomal groups exhibited a certain degree of tumor‐targeting, with the tumor and liver showing the highest copper content. Among them, the Cu‐Lip‐Gal(H) group had the highest copper content in the tumor, whereas copper content in other major organs was significantly decreased, demonstrating the strongest tumor‐targeting ability and the least impact on normal organs.

The results at 12 h and 48 h showed that copper content in the tumors of the Cu solution group decreased rapidly, compared with that at 0.5 h, whereas the liposomal groups maintained relatively high copper levels, demonstrating the sustained‐release advantage of liposomes. At 48 h, the copper content in the tumors of the Cu‐Lip‐Gal(H) group was approximately 40‐fold higher than that of the Cu solution group, suggesting a substantially improved therapeutic advantage for tumors.

### 2.9 Hematoxylin and Eosin (H&E)

To evaluate the in vivo toxicity of Cu^2+^, hearts, kidneys, livers, lungs, spleens, and tumors collected after the in vivo therapeutic efficacy study were stained with H&E. As shown in Figure 7A, histological analysis indicated that different treatments did not induce significant histopathological changes, compared with the control group, and no statistically significant differences were observed between groups. These results confirm that short‐term copper‐based treatment did not cause obvious structural damage to major organs in mice, thus providing preliminary evidence of its biological safety. However, in tumor tissues, the control group exhibited relatively intact cellular morphology; although nuclear atypia was present, fewer death‐related morphological changes were observed, suggesting a lower degree of cell death. In contrast, all other treatment groups exhibited varying degrees of nuclear abnormalities, with severity increasing from left to right. Among these, the Cu‐Lip‐Gal (H) group exhibited the most significant nuclear abnormalities, with prominent nuclear fragmentation and irregular structures, indicating the highest degree of cell death. The above results confirmed that Cu‐Lip‐Gal (H) could most effectively induce tumor cell death and inhibit tumor growth.

**FIGURE 7:**
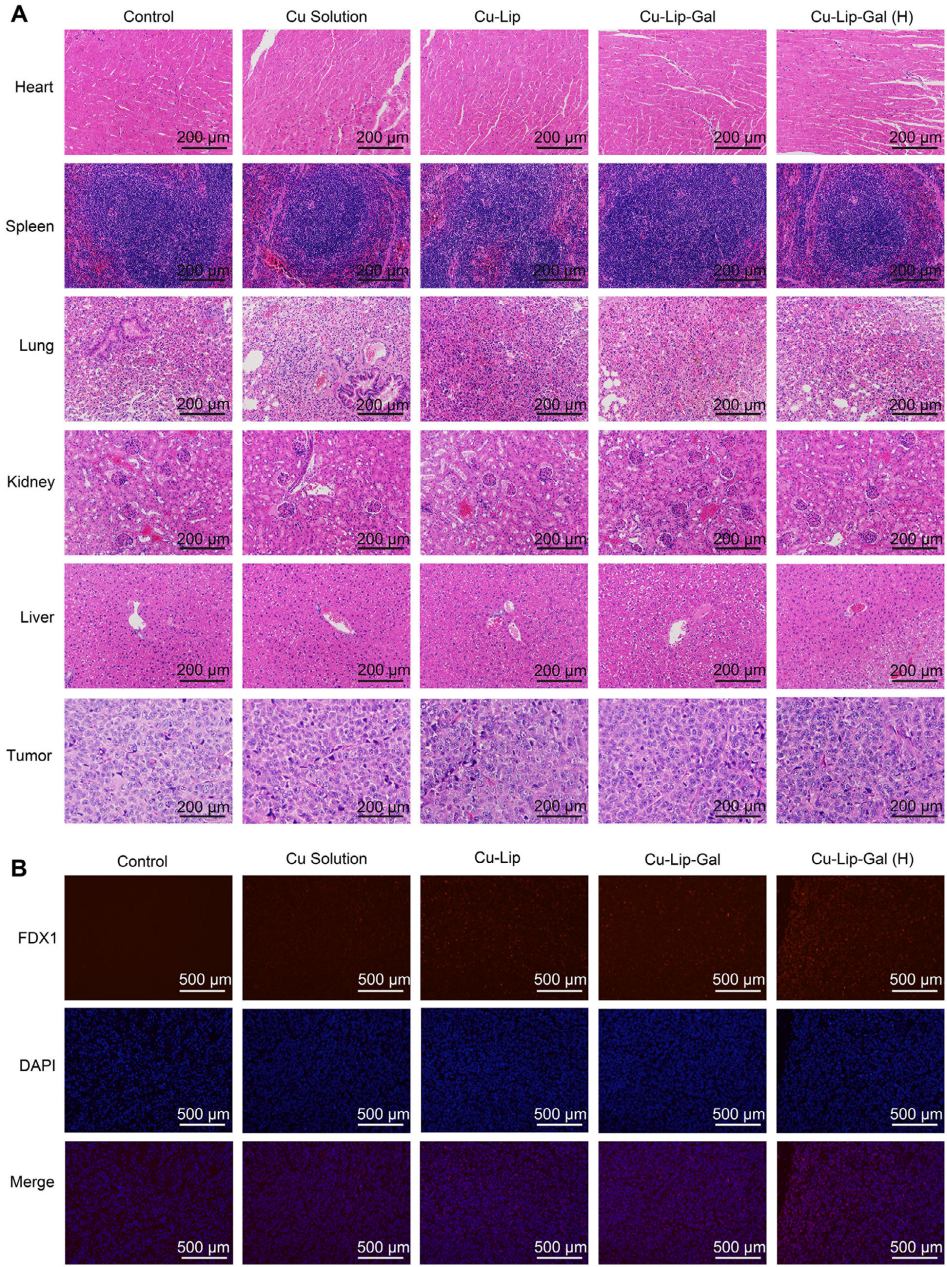
Histopathological analysis of major organs and FDX1 expression in tumor tissues. (A) H&E‐stained images of heart, spleen, lung, kidney, liver and tumor tissue. (B) The expression level of FDX1 in tumor tissue was detected by IF staining. Scale bar: × 200 × 500.

### 2.10 FDX1 Expression Level in Tumor Tissues

FDX1 promotes DLAT lipoylation and abates iron–sulfur cluster proteins by reducing Cu^2+^ to Cu^+^, inducing cell death [49]. Our WB and qPCR data revealed that the levels of FDX1 and DLAT were upregulated in HepG2 cells subjected to Cu‐Lip‐Gal (H), whereas these upregulations were synergistic outcome of the elevated intracellular copper content and HT, triggering the strongest cuproptosis in treatments. Immunofluorescence (IF) results (Figure 7B) of tumor tissues showed that from control to Cu‐Lip‐Gal (H) group, the expression of FDX1 increases gradually, indicating the strongest cuproptosis, which was in line with the findings of cell experiments and tumor H&E stain. The merged images (4',6‐diamidino‐2‐phenylindole [DAPI]/FDX1) further confirmed the cytoplasmic localization of FDX1, with minimal nuclear overlap, consistent with its role in mitochondrial iron–sulfur cluster biogenesis [50]. These findings collectively demonstrate that Cu‐Lip‐Gal(H) optimally induces FDX1‐mediated cuproptosis, providing a promising strategy for targeted cancer therapy.

## 3 Discussion

Cuproptosis is defined as a type of regulated cell death triggered by excessive intracellular copper accumulation, which disrupts copper homeostasis, impairs the TCA cycle, and ultimately leads to cell death [13]. However, the absence of targeted intervention strategies for intracellular copper homeostasis frequently elicits systemic adverse effects, which substantially impedes the clinical translation of cuproptosis in cancer therapy [14]. In this study, we developed Cu‐Lip‐Gal(H) for the targeted delivery of copper ions to HCC cells, which exhibited uniform particle size, regular spherical morphology, and distinct Tyndall effect, as well as excellent storage and physiological stability. The design of the lipid membrane composition of the liposome makes the phase transition temperature of the membrane 42°C, which displayed sustained copper release at 37°C and rapid release at 42°C. The delivery system simultaneously exploits the passive targeting effect of liposomes on solid tumors, the active targeting effect of galactose modification to the specific expressed ASGPR on hepatoma cells for anchoring, as well as the physicochemical targeting effect of local heating at the tumor site to promote thermosensitive release.

The cell viability of HepG2 and Huh7 cells treated with Cu‐Lip‐Gal(H) was approximately 24% and 18%, respectively, which was significantly lower than that of Cu‐Lip‐Gal and Cu‐Lip. This trend was consistent with the intracellular copper delivery efficiency. Mechanistically, Cu‐Lip‐Gal was internalized by HepG2 cells predominantly through ATP‐dependent clathrin‐mediated endocytosis. NADPH is the major cellular reductant for maintaining reduced GSH level and combating oxidative stress [51]. Its depletion severely impairs antioxidant defense capacity, exacerbates ROS‐mediated damage, and potentiates the proteotoxic cascade that is central to cuproptosis [51, 52]. Efficient copper delivery in the study resulted in a marked increase in intracellular ROS (up to 2.3‐fold of the control group) and a significant reduction in NADPH (approximately 67%), indicating severe redox imbalance and mitochondrial metabolic dysfunction. These changes were accompanied by the upregulated expression of core cuproptosis‐related proteins, FDX1 and DLAT, confirming the activation of copper‐dependent lipoylated protein aggregation and proteotoxic stress. In the colony formation ability and cell scratch assays, Cu‐Lip‐Gal(H) exerted the strongest inhibitory effect on colony formation and cell migration, indicating its potential to suppress tumor proliferation and metastasis. In vivo xenograft experiments further validated the strongest antitumor efficacy of Cu‐Lip‐Gal(H), with tumor inhibition rate of 86.7% ± 1.9%, and exhibited a significant increase in mice body weight, indicating that tumor progression was well controlled, and the mice maintained normal feeding and locomotor activity. Tissue distribution analysis revealed that Cu‐Lip‐Gal(H) significantly enhanced copper accumulation and retention in tumor tissues while reducing systemic copper exposure. At 0.5 h after administration, the tumor copper concentration in this group was 5.2‐fold that in the copper solution group; at 48 h post‐administration, the difference increased to about 40‐fold. In contrast, the copper solution group lacked targeting capacity, with copper mainly distributed in organs such as the liver and kidney. These results are consistent with previously reported findings on the targeted delivery of small‐molecule drugs using TSLs [53]. Histopathological analysis showed that none of the treatment groups induced histological damage to the major organs. However, the proportion of apoptotic cells in tumor tissues increased sequentially from the control group to the Cu‐Lip‐Gal(H) group. Furthermore, the IF expression trend of FDX1, a key marker of cuproptosis, was consistent with the apoptotic proportion, confirming that cuproptosis was the primary cause of tumor cell death after administration.

Notably, the HepG2 cell line used in this study is derived from human hepatoblastoma rather than clinical HCC. Although widely used as an in vitro HCC model due to its hepatic origin and similar biological characteristics, conclusions from HepG2 cells cannot be directly extrapolated to clinical HCC, and the translational potential requires further validation using patient‐derived primary HCC cells or organoids. Meanwhile, trace expression of ASGPR in liver non‐parenchymal cells may cause non‐specific drug uptake and potential copper accumulation in normal liver tissues. As a liposomal nanocarrier, Cu‐Lip‐Gal may be rapidly cleared by the mononuclear phagocyte system in vivo, and its long‐term colloidal stability, metabolic fate, and potential immunogenicity have not been systematically evaluated, which may affect its bioavailability and targeting efficiency. Additionally, the clinical translation of local HT is limited by challenges in precise temperature control, uniform heat distribution in solid tumors, and avoidance of thermal damage to normal tissues, requiring further optimization of technical feasibility and standardization. Meanwhile, these limitations point out clear directions for future research, namely, improving targeting specificity, enhancing liposome stability, optimizing HT regimens, and validating the therapeutic efficacy in clinically relevant HCC models.

## 4 Conclusion

In summary, this study presents a novel targeted copper ion delivery system, Cu‐Lip‐Gal(H), based on galactose‐modified TSLs, engineered to harness the cuproptosis mechanism for the treatment of HCC. Mechanistically, HT and elevated intracellular copper levels synergistically activate the FDX1/DLAT‐mediated cuproptosis pathway, accompanied by pronounced oxidative stress and mitochondrial metabolic dysfunction. In vivo experiments demonstrate that this strategy exerts a robust inhibitory effect on tumor growth, providing novel insights into the application of cuproptosis‐based copper ion delivery systems in HCC therapy.

## 5 Materials and Methods

### 5.1 Materials

1,2‐Dipalmitoyl‐sn‐glycero‐3‐phosphocholine (DPPC), 1‐stearoyl‐2‐hydroxy‐sn‐glycero‐3‐phosphocholine (MSPC), 1,2‐distearoyl‐sn‐glycero‐3‐phosphoethanolamine ‐N‐[methoxy(polyethylene glycol)‐2000] (DSPE‐PEG2000), and 1,2‐distearoyl‐sn‐glycero‐3‐phosphoethanolamine‐N‐[amino(polyethylene glycol)‐2000]‐N'‐galactosyl (DSPE‐PEG2000‐Gal) were obtained from Avanti Polar Lipids (Alabaster, AL, USA). Cell Counting Kit‐8 (CCK‐8; CK04, Beijing Huataixin Biomedical Technology Co. Ltd., Beijing, China). MTT Cell Proliferation and Cytotoxicity Assay Kit (SY0506, Beijing BioLab Scientific Co. Ltd., Beijing, China). Reactive Oxygen Species Assay Kit (S0033S, Beyotime Institute of Biotechnology, Shanghai, China). Dulbecco's modified eagle medium (DMEM) was purchased from Thermo Fisher Scientific (Waltham, MA, USA). Diethylpyrocarbonate (DEPC)‐treated water, PBS, and DAPI were sourced from Beijing Solebrite Technology Co. Ltd. (Beijing, China). NAC was purchased from Wako Pure Chemical Industries, Ltd. (Osaka, Japan). CS1 was obtained from Shanghai Yuanye Bio‐Technology Co. Ltd. (Shanghai, China).

### 5.2 Preparation of Copper‐Loaded Liposomes

Copper liposome (Cu‐Lip) and galactose‐modified liposome (Cu‐Lip‐Gal) were prepared via thin‐film hydration adapted from established TSL protocols [54]. DPPC, MSPC, and DSPE‐PEG2000/DSPE‐PEG2000‐Gal were used at an 85:10:5 molar ratio; they were dissolved separately in chloroform. The organic solvent was then removed under vacuum (40°C, 2 h), and the resulting lipid film was hydrated with 5 mL of CuCl_2_ solution (20 mM) at 60°C for 45 min. The suspensions were extruded through 0.22 µm polycarbonate membranes (10 cycles, 60°C) to yield Cu‐Lip and Cu‐Lip‐Gal.

### 5.3 Physicochemical Characterization and Stability

The initial macroscopic states of the Cu solution, Cu‐Lip, and Cu‐Lip‐Gal were observed with water as a control. Subsequently, the Tyndall effect was examined for each sample under both bright‐field and dark‐field conditions upon laser irradiation. The particle size distribution, PDI, and zeta potential were determined by dynamic light scattering (DLS) using a Malvern Zetasizer Nano ZS (UK). Morphological examination was performed using TEM (JEM‐1010, JEOL Ltd., Japan). The stability of the formulations was assessed under two distinct conditions. First, the storage stability of Cu‐Lip and Cu‐Lip‐Gal was evaluated at 4°C for 14 days by monitoring changes in particle size, PDI, and zeta potential. Second, to evaluate the colloidal stability under physiological‐like conditions, the particle size and PDI of Cu‐Lip‐Gal were recorded by DLS after incubation in PBS with 10% FBS for different times (1 day, 2 days, 3 days). To determine the retention of the encapsulated payload at these time points, the encapsulation efficiency (EE%) of copper ions was analyzed by dialyzing the aliquot against fresh PBS (pH 7.4) for 2 h to remove free copper, followed by liposome lysis with 1% (v/v) Triton X‐100 and copper quantification via inductively coupled plasma mass spectrometry (ICP‐MS). All measurements were performed in triplicate.

### 5.4 Determination of Cu^2+^ Content and Encapsulation Efficiency

Total Cu^2+^ content (C_total_) was quantified by lysing 200 µL of Cu‐Lip or Cu‐Lip‐Gal with 1% (v/v) Triton X‐100 and detecting by ICP‐MS (NexION 350X, PerkinElmer). The encapsulated Cu^2+^ content (C_encap_) was measured by ICP‐MS after dialysis in PBS (pH 7.4, 2 h), followed by identical lysis. All liposomal lysates were diluted 10‐fold in 2% HNO_3_ prior to ICP‐MS analysis. The encapsulation efficiency (EE%) was calculated as

$$\text{EE}\%=(C_{\mathrm{encap}}/C_{\mathrm{total}})\times 100\%.$$

### 5.5 Liposomes Disassembly and Cu^2+^ Release In Vitro

The Cu^2+^ release experiment was performed using the static Franz cell method. In brief, the regenerated cellulose membrane (MWCO: 1000 Da) was used to separate the donor and acceptor compartments, and the diffusion area was 2 cm^2^. The donor compartment was filled with 2 mL Cu‐Lip or Cu‐Lip‐Gal (20 µM). The receiving fluid was normal saline. Cu‐Lip and Cu‐Lip‐Gal were subjected to experiments at 37°C for 24 h to simulate their release in the blood circulation. For Cu‐Lip‐Gal (H), the experiment was initially conducted at 42°C for 0.5 h, after which the temperature was restored to 37°C; the total release duration was 24 h, aiming to mimic the in vivo release of TSLs. At predetermined time points, 0.5 mL of the receiving fluid was withdrawn to quantify Cu^2+^ via ICP‐MS. The cumulative amount of released Cu^2+^ was plotted against time (*n* = 3).

### 5.6 Cell Culture

The human hepatoblastoma cell line HepG2, HCC cell line Huh7, and human normal hepatocyte cell line LO2 were purchased from the Cell Resource Center (IBMS, CAMS/PUMC). All cell lines were authenticated by short tandem repeat (STR) profiling to verify their identity and confirmed to be free of mycoplasma contamination before use. All three cell lines were cultured in DMEM medium supplemented with 10% fetal bovine serum and 1% penicillin/streptomycin, and maintained at 37°C in a humidified incubator with 5% CO_2_.

### 5.7 In Vitro Cytotoxicity Assessment

Cell viability was measured using the standard MTT and CCK‐8 assays (*n* = 6). In detail, LO2 cells, HepG2 and Huh7 cells were seeded to 96‐well plates at a density of 1 × 10^4^ cells/well and 5 × 10^4^ cells/well, each containing 100 µL of culture medium. After 24 h standard culture at 37°C, the cells were treated with Cu‐Lip from 0.1 to 5 µM for 24 h. Next, cell viability was determined by the MTT assay. Subsequently, with the Cu^2+^ concentration set at 2.5 µM, MTT assays were performed on five groups: Control (PBS), Cu solution, Cu‐Lip, Cu‐Lip‐Gal and Cu‐Lip‐Gal (H). For Cu‐Lip‐Gal (H) group, after the addition of Cu‐Lip‐Gal, the 96‐well plate was sealed and subjected to a water bath heating at 42°C for 0.5 h.

For CCK‐8 proliferation kinetics, cells were treated identically above with measurements at 24, 36, and 48 h. At each endpoint, medium was replaced with 100 µL DMEM containing 10% CCK‐8 reagent. After 2 h of incubation at 37°C, plates were gently agitated for 5 min, and absorbance at 450 nm was recorded (Multiskan FC, Thermo Fisher). Cell viability was calculated as

$$\text{Cell}\text{viability}\left(\%\right)=\left(A_{\mathrm{treated}}-A_{\mathrm{blank}}\right)/\left(A_{\mathrm{control}}-A_{\mathrm{blank}}\right)\times 100\%$$

where A_blank_ represents cell‐free wells with CCK‐8 reagent.

### 5.8 Measurement of Intracellular Copper Ion

Intracellular copper ions were quantified using the Copper Detection Kit (Solarbio Life Sciences, Beijing, China). In short, cell or tissue homogenates were centrifuged at 10,000 × g for 10 min, and the supernatants were used for measurement of copper ion levels. The absorbance values were read on a microplate reader (BioTek Instruments, Winooski, VT, USA).

### 5.9 Cellular Uptake

HepG2 cells were seeded in confocal dishes and treated with chlorpromazine, colchicine or 4°C. Subsequently, the cells were treated with Coumarin‐6‐labeled Cu‐Lip‐Gal (Cu‐Lip‐Gal/Cou‐6). After incubation for 4 h, cells were washed with PBS, fixed at room temperature for 10 min, and stained with DAPI for 5 min. Afterward, the fluorescence intensity of Coumarin‐6 was monitored with CLSM (Nikon Corporation, Tokyo, Japan).

### 5.10 Measurement of Intracellular Cu^+^ Level by Coppersensor‐1 (CS1) Probe

To clarify the key role of FDX1‐mediated Cu^2^^+^ reduction to Cu^+^ in cuproptosis, intracellular Cu^+^ levels were specifically detected using the CS1 probe. HepG2 cells were seeded in confocal dishes at 2 × 10^5^ cells/dish, cultured at 37°C with 5% CO_2_ for 24 h to full adherence, then divided into three groups and treated with Coumarin‐6 (COU‐6)‐labeled Cu‐Lip, Cu‐Lip‐Gal, and Cu‐Lip‐Gal(H) at a final Cu^2^^+^ concentration of 2.5 µM. For the Cu‐Lip‐Gal(H) group, HT was performed at 42°C for 0.5 h immediately after dosing, followed by further incubation at 37°C for 4 h. After treatment, the culture medium was removed, and cells were washed three times with pre‐warmed PBS (pH 7.4) to eliminate unbound Cu^2^^+^ and free COU‐6. Cells were then incubated with 5 µM CS1 probe solution at 37°C for 30 min in the dark to ensure sufficient probe penetration and specific binding to intracellular Cu^+^ [55] with excess unbound probe removed by three additional PBS washes. Subsequently, cells were fixed with 4% paraformaldehyde at room temperature for 10 min, rinsed three times with PBS, and stained with DAPI for 5 min in the dark to label nuclei. After washing off excess DAPI with PBS, cells were immediately imaged via CLSM.

### 5.11 Intracellular NADPH/ROS Levels Measurement and Oxidative Stress Intervention Experiment

The HepG2 cells (2 × 10^5^/well) were inoculated in 6‐well plates. Then the cells were treated with different samples for 4 h: (1) control (fresh DMEM), (2) Cu solution, (3) Cu‐Lip, (4) Cu‐Lip‐Gal, (5) Cu‐Lip‐Gal(H). For Cu‐Lip‐Gal (H) group, the plate should be sealed and heated for 0.5 h as before. Then the cytosolic level of NADPH was quantified by NADPH determination assay kit (Biovision, San Francisco, CA, USA). For intracellular ROS level detection, the HepG2 cells (2 × 10^5^/well) were grown in confocal dishes overnight. The cells were then exposed to five treatments as above. After 4 h, the cells were washed with PBS, and the medium was replaced by a fresh one, followed by cell staining with a DCFH‐DA probe (10 µM) and incubation for another 30 min at 37°C without light. Then, the cells were imaged by CLSM with the excitation and emission wavelength at 488/530 nm. To verify the causal role of oxidative stress in Cu‐Lip‐Gal(H)‐induced cuproptosis, NAC was used as an ROS scavenger, with three experimental groups established: Control (fresh DMEM), Cu‐Lip‐Gal(H) group (treated with 2.5 µM Cu^2^^+^ followed by HT at 42°C for 0.5 h), and NAC + Cu‐Lip‐Gal(H) group (preincubated with 5 mM NAC for 2 h prior to the same treatment as the Cu‐Lip‐Gal(H) group). At 4 h post‐treatment, intracellular ROS levels were detected via DCFH‐DA staining (as described above) with fluorescence intensity quantified using ImageJ software, while intracellular NADPH levels were determined with the NADPH Assay Kit (results expressed as fold change relative to the control group); cell viability was evaluated by MTT and CCK‐8 assays after 24 h of treatment. All experiments were performed in triplicate (*n* = 3).

### 5.12 Colony Formation and Scratch Assays

In a colony formation assay, HepG2 cells (1 × 10^4^/well) were plated in 6‐well plates. The following day, attached cells were treated with PBS (control), Cu solution, Cu‐Lip, Cu‐Lip‐Gal, and Cu‐Lip‐Gal (H). The plate of Cu‐Lip‐Gal (H) was sealed and heated for 0.5 h as before. After 12 h, the medium was replaced with fresh culture medium to allow colony formation. One week later, fixed colonies were stained with crystal violet solution (Beyotime, China; C0121), imaged using a microscope (Olympus, Japan; IX71), and counted with ImageJ software (National Institutes of Health, USA).

In the scratch assay, cells were cultured in 6‐well plates to 100% confluence and scratched. Then, the detached cells were washed with serum‐free medium. The remaining cells were treated with PBS (control), Cu solution, Cu‐Lip, Cu‐Lip‐Gal, and Cu‐Lip‐Gal (H) for 12 h as before, followed by medium replacement with serum‐free DMEM. After 12, 24, 36, and 48 h of incubation, images of the scratch were taken, and the healing area was quantified using ImageJ software.

### 5.13 Western Blot Analysis

To investigate the cuproptosis mechanism of Cu loaded liposomes and HT, we analyzed the expression levels of key proteins (FDX1, DLAT) [14, 46] in five treatment groups: control (PBS), Cu solution, Cu‐Lip, Cu‐Lip‐Gal, and Cu‐Lip‐Gal(H). Cells were lysed in a RIPA lysis buffer (Beyotime, China; P0013B) supplemented with protease and phosphatase inhibitors (Beyotime, China; P1050). Proteins were extracted and denatured. Equal amounts of protein (20 µg) were separated on a 10% SDS‐PAGE gel (Beyotime, China; P0455M) and transferred to polyvinylidene fluoride (PVDF) membranes (Millipore, Germany; IPVH00010). Membranes were blocked with 5% bovine serum albumin (BSA) in TBST to prevent nonspecific binding, then incubated with primary antibodies. Primary antibodies included anti‐β‐tubulin (Affinity Biosciences, China; T0023), anti‐DLAT (4A4‐B6‐C10) and anti‐FDX1 (ab108257) from Cell Signaling Technology (USA). Protein bands were visualized using corresponding horseradish peroxidase (HRP)‐conjugated secondary antibodies and an enhanced chemiluminescence (ECL) system (Beyotime, China; P0018FM), and semi‐quantitative analysis was conducted with Odyssey imaging system (Li‐Cor Biosciences).

### 5.14 Quantitative Real‐Time Polymerase Chain Reaction (RT‐qPCR)

Total RNA was extracted from HepG2 cells using an RNA extraction kit (Promega, USA; LS1040) following the manufacturer's protocol. Equal amounts of RNA were reverse‐transcribed into first‐strand cDNA using a reverse transcription kit (Promega, USA; A2800). Quantitative polymerase chain reaction (qPCR) was performed to assess mRNA expression using the Go Taq qPCR and RT‐qPCR Systems (Promega, USA; A6001). Primer sequences are listed in Table S1, and β‐actin served as the internal control for mRNA quantification. Specificity of amplification was confirmed by melting curve analysis. Relative expression levels of mRNAs were calculated using the 2^(−ΔΔCt)^ method.

### 5.15 Animal Model

Female nu/nu nude mice (4–6 weeks old, 18–22 g) were purchased from Vital River Laboratories (Beijing, China). All animals were housed under specific pathogen‐free (SPF) conditions at 22°C ± 2°C with a 12 h light/dark cycle and free access to sterile food and water. A xenograft tumor model was produced via subcutaneous injection of 100 µL HepG2 cells solution containing 1 × 10^6^ cells. In the in vivo anti‐tumor study, 30 mice with uniform tumor volumes (480 ± 20 mm^3^) were randomly divided into five groups (*n* = 6 per group). For tissue distribution analysis, an additional 36 tumor‐bearing mice were randomly assigned to four groups (*n* = 9 per group). All experimental procedures were approved by the Animal Protection Ethics Committee of Tianjin University of Science and Technology (Approval No. tust‐ec‐2025.1.2) and were conducted in strict accordance with the ARRIVE 2.0 guidelines.

### 5.16 In Vivo Antitumor Efficacy Evaluation

30 nude mice bearing similar sized tumors (initial tumor volume: 480 ± 20 mm^3^) were selected from HepG2 tumor‐bearing mice, then randomly divided into five groups (*n* = 6): (1) control (saline, 0.9% NaCl), (2) Cu solution, (3) Cu‐Lip, (4) Cu‐Lip‐Gal, and (5) Cu‐Lip‐Gal (H). The tumor‐bearing mice received four intravenous (i.v.) injections via tail vein at 4‐day intervals (on Days 0, 4, 8, and 12 of the treatment period). HT was applied to the Cu‐Lip‐Gal (H) group using an infrared lamp (Philips IR250, 250W) focused on tumors for 0.5 h at 42°C ± 0.5°C immediately post‐injection, monitored by infrared thermography (FLIR E60) [56, 57]. Tumor dimensions were measured every other day from Day 0 (treatment initiation) to Day 14 using a digital caliper. At the end of the study, the mice were euthanized, and the excised tumors were carefully trimmed of adherent connective tissue, fat pads, and skin before weighing and photography. The other main organs (heart, liver, spleen, lung, kidney) were also collected for following H&E stain and IF stain for tumor.

The formula for calculating tumor volume is as follows:

$$V=\frac{1}{2}\times L\times S^{2}$$

V: tumor volume; L: longest diameter of the tumor (long axis); S: shortest perpendicular diameter of the tumor (short axis).

The formula for calculating the tumor inhibition rate is as follows:

$$\text{IR}\left(\%\right)=\left(1-m_{\mathrm{tx}}/m_{\mathrm{ctrl}}\right)\times 100\%$$

IR: tumor inhibition rate; m_tx_: average tumor weight of the experimental group; m_ctrl_: average tumor weight of the control group.

### 5.17 Biodistribution Study

To further investigate the effects of passive targeting by liposomes, active targeting by galactose modification for hepatocytes, and physicochemical targeting by local tumor HT on the in vivo distribution of Cu, 36 HepG2 tumor‐bearing mice with tumor sizes approximately 1000 mm^3^ were randomly divided into four groups (*n* = 9): Cu Solution, Cu‐Lip, Cu‐Lip‐Gal, and Cu‐Lip‐Gal(H). The mice received i.v. injections via tail vein, and the Cu‐Lip‐Gal(H) group was subjected to 0.5 h of tumor local HT as before. At 0.5, 12, and 48 h post administration, three mice per group were sacrificed. Subsequently, the collected tissue samples (heart, liver, spleen, lung, kidney, tumor) were homogenized and centrifuged, and the supernatant was detected by method 2.8.

### 5.18 H&E

The organs collected at 5.15. section were fixed with 4% paraformaldehyde solution and processed by dehydration, paraffin embedding, and sectioning (5 µm). The slices were dewaxed and then washed with different concentrations of ethanol and distilled water; stained in hematoxylin, rinsed in tap water for 2 min, and stained in eosin. Glass slides were further rinsed in tap water and dehydrated in increasing concentrations of ethanol ending in xylene and coverslip using pertex [58]. Glass slides were analyzed by light microscopy to assess overall tissue integrity.

### 5.19 IF Stain

IF was used to detect FDX1 in mice tumors collected at 2.15. section [50, 59]. Briefly, 5 µm tumor sections were dewaxed and rehydrated. Subsequently, samples were permeabilized with 0.1% Triton‐X and rinsed three times with PBS. The tumors were then blocked with 5% BSA for 30 min and incubated overnight at 4°C with primary antibodies against anti‐FDX1 (55110‐1‐AP, Proteintech). Then, the samples were incubated with a fluorescence‐coupled secondary antibody (Invitrogen) for 1 h at 25°C. After that, DAPI staining was utilized to dye nuclei for 10 min. Imaging was conducted using the fluorescence microscope (Zeiss, Germany).

### 5.20 Statistical Analysis

Statistical analyses were performed using GraphPad Prism 9.0 (GraphPad Software, San Diego, CA). Quantitative data are presented as mean ± standard deviation (SD). Group comparisons were analyzed by unpaired Student's *t*‐test (for two groups) or one‐way/two‐way ANOVA with Tukey's post hoc test (for multiple groups). Statistical significance thresholds were designated as follows: n.s. no significant difference, *p < 0.05, **p < 0.01, ***p < 0.001. For datasets requiring pairwise comparisons, significant differences (*p* < 0.05) are additionally indicated by superscript letters (a, b, c), where groups sharing the same letter do not differ significantly.

## Author Contributions

H.Y. curated the data, performed formal analysis, conducted the investigation, developed the methodology, developed the software, validated the results, prepared the visualizations, drafted the original manuscript, and reviewed and edited the manuscript. H.H. curated the data, performed formal analysis, conducted the investigation, developed the software, prepared the visualizations, and reviewed and edited the manuscript. H.L. conceptualized the study, curated the data, performed formal analysis, conducted the investigation, developed the software, prepared the visualizations, and reviewed and edited the manuscript. X.L.W. conducted the investigation, curated the data, performed formal analysis, developed the software, and drafted the original manuscript. W.Z. conducted the investigation, curated the data, performed formal analysis, and reviewed and edited the manuscript. M.Y. conducted the investigation, curated the data, and prepared the visualizations. F.Y.Z. conducted the investigation and prepared the visualizations. F.X.Z. curated the data and prepared the visualizations. M.L. prepared the visualizations. X.C.W. administered the project, provided the resources, and supervised the study. X.G.Z. conceptualized the study, performed formal analysis, acquired the funding, conducted the investigation, developed the methodology, administered the project, provided the resources, supervised the study, validated the results, and reviewed and edited the manuscript. All authors have read and approved the final manuscript.

## Funding

This study was supported by National Natural Science Foundation of China (82660635, 82560042); Central guiding local technology development projects (2024ZY0152); Inner Mongolia Autonomous Region science and technology planning project (2026YFSH0155, 2026YFHH0127, 2023YFSH0029, 2025YFSH0060); Natural Science Foundation of Inner Mongolia (2025ZDLH019, 2025MS08012, 2024QN08075, 2024LHMS08049); Clinical medical research and clinical new technology promotion projects of Inner Mongolia Autonomous Region Health Commission (YSXH2024KYF065); Science and Technology Program of the Joint Fund of Scientific Research for the Public Hospitals of Inner Mongolia Academy of Medical Sciences(2023GLLH0138, 2023GLLH0007, 2023GLLH0136, 2023GLLH0142, 2023GLLH0139); Science and Technology Project of Inner Mongolia Autonomous Region Health Commission (2025NMWJKJXM0255); Shenzhen Basic Research Special Program (Natural Science Foundation) – General Program (JCYJ20240813103804007); Zhi Yuan Talent Projects of Inner Mongolia Medical University (ZY20241213); Key Specialist Discipline Construction Project (Radiotherapy & Pathology) (2025YNFL001, 2025YNFL005); National University Students' Innovation and Entrepreneurship Training Program (202610132010S).

## Ethics Statement

All animal procedures were approved by the Animal Protection Ethics Committee of Tianjin University of Science and Technology (Approval No. tust‐ec‐2025.1.2) and strictly followed the ARRIVE 2.0 guidelines and international animal welfare standards. Female SPF‐grade nu/nu nude mice (4–6 weeks, 18–22 g) were purchased from Vital River Laboratories (Beijing, China) and housed under standard SPF conditions with ad libitum access to sterile food and water. The initial tumor volume was set at approximately 500 mm^3^ (480 ± 20 mm^3^) to ensure uniform tumor burden across groups. Mice received four i.v. injections at 4‐day intervals and were humanely euthanized on Day 14 (48 h after the final dose) to evaluate the complete therapeutic effect of TSLs, as sufficient time is required for nanoparticle accumulation via the EPR effect and full manifestation of cuproptosis. Due to this scientific necessity, tumor volumes in the untreated blank control group and free copper solution group may slightly exceed 2000 mm^3^ at the endpoint. However, all tumor weights were strictly controlled below 10% of body weight, and no signs of pain or abnormal behaviors were observed in any mice with twice‐daily health monitoring. This experimental design and the slight volume exceedance have obtained special approval from the ethics committee.

## Conflicts of Interest

The authors declare no conflicts of interest.

## Supporting information




**Supporting File**: mco271068‐sup‐0001‐SuppMat.docx

## Data Availability

The data supporting this work are accessible upon reasonable request from the corresponding author.
