# Supplementary material for: Hepatocellular Carcinoma Targeted Thermosensitive Cu2+ Liposomes Based on the Cuproptosis Mechanism
Source: MedComm (2020). 2026 Oct 13;7(10):e71068. doi: 10.1002/mco2.71068 (PMC13659279; doi:10.1002/mco2.71068)
Supplement: Supplementary file 1 [file MCO2-7-e71068-s001.docx]

**Supplemental Information**

Corresponding author:

Xiangcheng Wang, Ph.D. Prof.

E-mail: lmynano@tust.edu.cn

Xianggao Zhu, Ph.D. Prof.

E-mail: yanghao0135@immu.edu.cn

**Hepatocellular Carcinoma Targeted** **Thermosensitive Cu^2+^ Liposomes Based on the Cuproptosis Mechanism**

Hao Yang^1, #^, Hui Hou^2,3, #^, Hong Li^1^, Xianglong Wang^4^, Wei Zhang^1^, Mengsi Yin^4^, Fuyu Zhang^1^, Faxin Zhang^4^, Mingyuan Li^4^, Xiangcheng Wang^5, *^, Xianggao Zhu^1, *^

\# Co-first authors

* Corresponding authors

1 Department of Radiation Oncology, Peking University Cancer Hospital (Inner Mongolia Campus) & Affiliated Cancer Hospital of Inner Mongolia Medical University, Inner Mongolia Autonomous Region, China

2 Department of Pediatric, Maternal and Child Health Care Hospital of the Inner Mongolia autonomous region, Inner Mongolia Autonomous Region, China

3 Department of Pediatric Hematology and Oncology, Inner Mongolia Autonomous Region People’s Hospital, Inner Mongolia Autonomous Region, China

4 College of Biotechnology, Tianjin University of Science & Technology, Tianjin, China

5 Department of Nuclear Medicine, Shenzhen People's Hospital, Shenzhen, China

**Supplemental information**

1. **Supplemental figures**
2. **Supplemental table**

**Figure S1.** The storage stability of nanoparticles over time under simulated physiological conditions. (A) The size distribution. (B) The PDI. (C) The zeta potential. (D) The EE of Cu-Lip and Cu-Lip-Gal.

**Figure S2.** Cell viability of LO2. (A) MTT assay; (B) CCK8 assay. (C) The LO2 cell growth curves after treatments in 48 h.

**Figure S3.** (A) The levels of reactive oxygen species (ROS) in HepG2 and Huh7 cells after NAC treatment. (B) The levels of reduced nicotinamide adenine dinucleotide phosphate (NADPH) in HepG2 and Huh7 cells after NAC treatment. (C) MTT assay; (D) CCK8 assay.

**Table S1.** Primer sequences used in qRT-PCR.

| Primers | Primer sequences |
| --- | --- |
| FDX1 |  |
| Forward: | 5'- TTCAACCTGTCACCTCATCTTTG -3' |
| Reverse: | 5'- TGCCAGATCGAGCATGTCATT -3' |
| DLAT |  |
| Forward: | 5'- TCATCTGTATCACAGTTGAAAAGC -3' |
| Reverse: | 5'- GGAAGAACAATCTGCATATGAGTAG -3' |
